# Correlation of the systemic immune-inflammation index with short- and long-term prognosis after acute ischemic stroke

**DOI:** 10.18632/aging.204228

**Published:** 2022-08-19

**Authors:** Nan Wang, Yingying Yang, Baoshan Qiu, Ying Gao, Anxin Wang, Qin Xu, Xia Meng, Yuming Xu, Bo Song, Yongjun Wang, Yilong Wang

**Affiliations:** 1 Department of Neurology, The First Affiliated Hospital of Zhengzhou University, Henan, China; 2 Department of Neurology, Beijing Tiantan Hospital, Capital Medical University, Beijing, China; 3 China National Clinical Research Centre for Neurological Diseases, Beijing, China

**Keywords:** thrombus inflammation, stroke, immunity, inflammation, prognosis

## Abstract

Immune and inflammatory responses after stroke are important pathophysiological processes. This study explored the relationship between the systemic immune-inflammation index (SII) and stroke prognosis. Patients from the China National Stroke Registry III were investigated. SII was defined as neutrophils × platelets/lymphocytes, and the patients were divided into four groups according to quartiles based on SII values. The primary outcome was poor functional outcome, assessed by the modified Rankin Scale (mRS), defined as an mRS score of ≥3. The secondary outcome was the incidence of all-cause death and recurrent stroke. Data were analyzed using either the logistic regression or Cox regression models. As the SII quartile increased, the percentage of patients with poor functional outcomes increased: 178 (7.8%), 223 (9.8%), 292 (12.8%), and 417 (18.3%) (*P* < 0.0001) at the 90-day follow-up and 172 (7.6%), 203 (8.9%), 266 (11.7%), and 386 (17.0%) (*P* < 0.0001) at the 1-year follow-up. Compared to patients in the quartile (Q)1 group, those in the Q4 group had a higher risk for adverse events, especially all-cause death at the 90-day follow-up (adjusted hazard ratio [HR], 2.409; 95% confidence interval [CI], 1.273–4.559, *P* = 0.0069) and at the 1-year follow-up visits (adjusted HR, 2.209; 95% CI, 1.474–3.311, *P* = 0.0001). The SII was closely related to the short- and long-term prognosis of patients with acute ischemic stroke, and patients with higher SIIs were more likely to have poor outcomes.

## INTRODUCTION

Stroke is the leading cause of death among the Chinese, and ischemic stroke accounts for approximately 82% of cases [1]. The immune and inflammatory responses after an acute ischemic stroke are complex pathophysiological processes that affect the prognosis of the disease [2]. Researchers have described how inflammatory immune cells (neutrophils, T cells, platelets, dendritic cells, monocytes, and/or macrophages) infiltrate the brains of patients with stroke [3, 4]. There are well-known correlations between stroke and a number of common biological indices, such as the neutrophil/lymphocyte ratio and the platelet/lymphocyte ratio. However, new indices are necessary to fully assess the prognosis of patients with acute ischemic stroke.

The systemic immune-inflammation index (SII) is a new type of comprehensive inflammation index that reflects the balance of the host’s immune and inflammatory states. The index is defined as neutrophils × platelets/lymphocytes [5]. The SII was first proposed in liver cancer research, aiming to identify patients with a high risk of recurrence or death, so early intervention could be initiated [5, 6]. In recent years, research on thrombus inflammation has attracted increasing attention. It is known that the interaction of platelets and neutrophils drives the occurrence and development of inflammation and damages brain tissues [7]. To date, cross-sectional studies have explored the relationship between the SII and stroke. One of these studies showed that the SII is closely related to the severity of acute ischemic stroke on admission [8]. However, prospective cohort studies exploring this relationship in patients with ischemic stroke are rare. A multicenter, large sample cohort study with a long follow-up is particularly lacking.

Therefore, we hypothesized that the prognosis of patients with acute ischemic stroke is related to the SII. In this study, we aimed to explore the relationship between the SII and the short- and long-term prognosis of patients who had an acute ischemic stroke.

## RESULTS

### Baseline characteristics

A total of 9,107 patients from the China National Stroke Registry III (CNSR-III) qualified for this study (Supplementary Figure 1 and Supplementary Table 1). The average age of the enrolled patients was 61.9 ± 11.1 years (Table 1). These patients were divided into four groups according to the SII quartile: quartile (Q)1, <366 × 10^9^/L; Q2, 366–533 × 10^9^/L; Q3, 534–799 × 10^9^/L; and Q4, ≥800 × 10^9^/L. The median time from symptom onset to admission was 10 (interquartile range [IQR], 3–25) h. For all patients, the median National Institutes of Health Stroke Scale (NIHSS) score at admission was 3 (IQR, 2–6) points. To exclude an effect of the pre-admission modified Rankin Scale (mRS) score on prognosis, the lack of significant group differences regarding the percentages of mRS scores before onset ≥3 was confirmed (*P* = 0.0603).

**Table 1:** Baseline characteristics of patients stratified by SII quartile.

**Variable**	**Total (*n* = 9,107)**	**SII quartile**				***P* value**
		**Q1 (*n* = 2,269)**	**Q2 (*n* = 2,285)**	**Q3 (*n* = 2,279)**	**Q4 (*n* = 2,274)**	
**Demographic and clinical features**						
Age (years), mean ± SD	61.9 ± 11.1	62.7 ± 10.7	61.4 ± 10.9	61.5 ± 11.3	61.9 ± 11.4	0.0004
Sex, Male, *n* (%)	6,343 (69.7)	1,566 (69.0)	1,651 (72.3)	1,589 (69.7)	1,537 (67.6)	0.0063
Smoker, *n* (%)	3,317 (36.4)	830 (36.6)	891 (39.0)	842 (37.0)	754 (33.2)	0.0006
Drinking, *n* (%)	3,553 (39.0)	867 (38.2)	945 (41.4)	921 (40.4)	820 (36.1)	0.0011
**Medical history**						
Ischemic stroke, *n* (%)	1,850 (20.3)	424 (18.7)	468 (20.5)	479 (21.0)	479 (21.1)	0.1550
Coronary heart diseases, *n* (%)	914 (10.0)	244 (10.8)	216 (9.5)	215 (9.4)	239 (10.5)	0.3036
Atrial fibrillation, *n* (%)	592 (6.5)	166 (7.3)	136 (6.0)	140 (6.1)	150 (6.6)	0.2480
Hypertension, *n* (%)	5,717 (62.8)	1,346 (59.3)	1,440 (63.0)	1,444 (63.4)	1,487 (65.4)	0.0003
Diabetes mellitus, *n* (%)	2,126 (23.3)	559 (24.6)	542 (23.7)	559 (24.5)	466 (20.5)	0.0024
Hypercholesterolemia, *n* (%)	696 (7.6)	167 (7.4)	191 (8.4)	175 (7.7)	163 (7.2)	0.4492
Hours of event onset, median (IQR)	10 (3–25)	10 (3–26)	11 (3–26)	11 (3–26)	8 (3–24)	<0.0001
NIHSS score at admission, median (IQR)	3 (2–6)	3 (1–5)	3 (2–5)	3 (2–6)	4 (2–7)	<0.0001
mRS score before onset ≥3, *n* (%)	370 (4.1)	82 (3.6)	84 (3.7)	90 (4.0)	114 (5.0)	0.0603
**Laboratory tests**						
WBC (10^9^/L)	7.24 ± 2.21	6.08 ± 1.60	6.70 ± 1.67	7.28 ± 1.75	8.90 ± 2.59	<0.0001
FPG (mmol/L)	6.46 ± 2.62	6.30 ± 2.47	6.41 ± 2.67	6.51 ± 2.58	6.63 ± 2.77	<0.0001
LDL-C (mmol/L)	2.56 ± 1.04	2.48 ± 0.99	2.52 ± 0.97	2.57 ± 1.06	2.68 ± 1.12	<0.0001
Hcy (μmol/L)	18.93 ± 12.26	18.36 ± 11.41	18.98 ± 12.94	18.78 ± 11.99	19.61 ± 12.62	0.0206
hs-CRP (mg/L)	5.67 ± 18.22	3.41 ± 10.23	4.35 ± 12.82	5.23 ± 15.49	9.62 ± 28.11	<0.0001
**90-day follow-up**						
Poor functional outcome, *n* (%)	1,110 (12.2)	178 (7.8)	223 (9.8)	292 (12.8)	417 (18.3)	<0.0001
Recurrent stroke, *n* (%)	534 (5.9)	127 (5.6)	98 (4.3)	139 (6.1)	170 (7.5)	<0.0001
All-cause death, *n* (%)	85 (0.9)	13 (0.6)	14 (0.6)	18 (0.8)	40 (1.8)	<0.0001
**1-year follow-up**						
Poor functional outcome, *n* (%)	1,027 (11.3)	172 (7.6)	203 (8.9)	266 (11.7)	386 (17.0)	<0.0001
Recurrent stroke, *n* (%)	847 (9.3)	198 (8.7)	182 (8.0)	206 (9.0)	261 (11.5)	0.0003
All-cause death, *n* (%)	205 (2.3)	34 (1.5)	38 (1.7)	45 (2.0)	88 (3.9)	<0.0001

Abbreviations: SII: systemic immune-inflammation index; SD: standard deviation; IQR: interquartile range; NIHSS: National Institutes of Health Stroke Scale; Q: quartile; mRS: modified Rankin Scale; WBC: white blood cell; FPG: fasting plasma glucose; LDL-C: low-density lipoprotein cholesterol; Hcy: homocysteine; hs-CRP: high-sensitivity C-reactive protein.

### SII and functional outcome

Table 1 displays the data for patients with poor functional outcomes at the 90-day and 1-year follow-up. Overall, 1,110 (12.2%) and 1,027 (11.3%) patients had poor functional outcome at the 90-day and 1-year follow-up, respectively. With the increase in SII, whether follow-up at 90-day or 1-year, the number of patients with poor functional outcomes was increasing.

**Figure 1:**
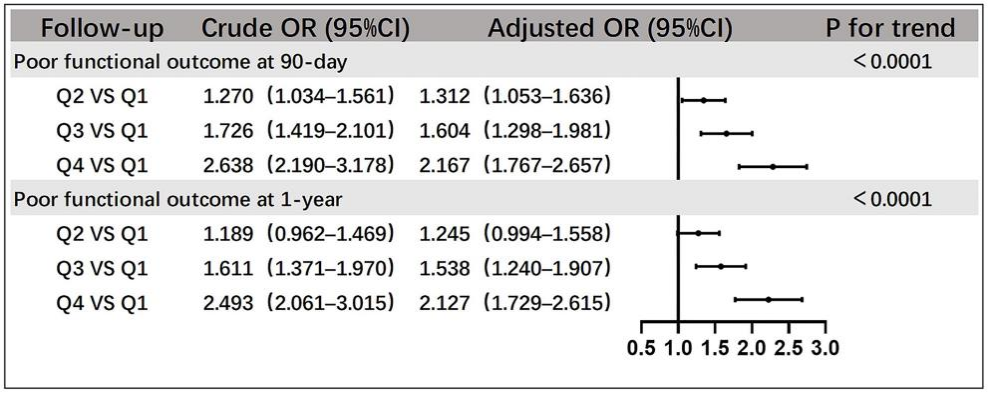
**Relationships between SII quartiles and poor functional outcomes of patients with acute ischemic stroke at the 90-day and 1-year follow-up.** Adjustment factors include sex, age, smoking status, alcohol consumption, history of cerebral infarction, hypertension, atrial fibrillation, coronary heart disease, diabetes mellitus, WBC, FPG, LDL-C, Hcy, hs-CRP, hours of event onset, NIHSS score at onset, and mRS score before onset ≥3. Abbreviations: WBC: white blood cell; FPG: fasting plasma glucose; LDL-C: low-density lipoprotein; Hcy: homocysteine; hs-CRP: high-sensitivity C-reactive protein.

The relationship between SII and poor functional outcome of stroke, analyzed using logistic regression, is shown using a forest plot (Figure 1). At the 90-day follow-up, the crude odds ratios (ORs) of Q2, Q3, and Q4 compared with Q1 were 1.270 (95% confidence interval [CI], 1.034–1.561), 1.726 (95% CI, 1.419–2.101), and 2.638 (95% CI, 2.190–3.178), respectively. At the 1-year follow-up, the crude ORs were 1.189 (95% CI, 0.962–1.469), 1.611 (95% CI, 1.371–1.1970), and 2.493 (95% CI, 2.061–3.015) for Q2, Q3, and Q4, respectively. After adjusting for confounding factors, the ORs of Q2, Q3, and Q4 compared with Q1 were 1.312 (95% CI, 1.053–1.636), 1.604 (95% CI, 1.298–1.981), and 2.167 (95% CI, 1.767–2.657) at the 90-day follow-up, and at 1-year follow-up, they were 1.245 (95% CI, 0.994–1.558), 1.538 (95% CI, 1.240–1.907), and 2.127 (95% CI, 1.729–2.615), respectively. The trend test revealed statistical significance (*P* < 0.0001). In addition, we conducted a stratified analysis based on the patients’ age, sex, smoking status, and alcohol consumption to further understand the effects of SII on the functional prognosis in different populations (Table 2). However, a particular subpopulation was not identified (*P* ≥ 0.10 for all interactions).

**Table 2:** Stratified analysis of the relationship between SII quartiles and risk of poor functional outcome.

		**Q1**	**Q2**	**Q3**	**Q4**	**P for trend**	**P for interaction**
**Poor functional outcome at 90-day follow-up**							
Age	<50 years, adjusted OR (95% Cl)	1 (ref)	1.930 (0.800–4.655)	1.950 (0.830–4.591)	2.650 (1.135–6.189)	0.0319	0.7111
	≥50 years, adjusted OR (95% Cl)	1 (ref)	1.235 (0.984–1.550)	1.542 (1.240–1.918)	2.104 (1.706–2.594)	<0.0001	
Sex	Male, adjusted OR (95% Cl)	1 (ref)	1.266 (0.961–1.668)	1.583 (1.213–2.064)	2.113 (1.633–2.735)	<0.0001	0.9856
	Female, adjusted OR (95% Cl)	1 (ref)	1.406 (0.972–2.034)	1.638 (1.154–2.324)	2.231 (1.597–3.118)	<0.0001	
Smoking	Smoking, adjusted OR (95% Cl)	1 (ref)	1.691 (1.125–2.543)	2.246 (1.511–3.339)	2.326 (1.563–3.463)	<0.0001	0.1084
	Does not smoke, adjusted OR (95% Cl)	1 (ref)	1.186 (0.910–1.545)	1.395 (1.084–1.795)	2.127 (1.675–2.701)	<0.0001	
Drinking	Drinking, adjusted OR (95% Cl)	1 (ref)	1.149 (0.783–1.687)	1.846 (1.286–2.649)	2.307 (1.614–3.299)	<0.0001	0.1625
	Does not drink, adjusted OR (95% Cl)	1 (ref)	1.410 (1.076–1.848)	1.484 (1.142–1.928)	2.102 (1.639–2.697)	<0.0001	
**Poor functional outcome at 1-year follow-up**							
Age	<50 years, adjusted OR (95% Cl)	1 (ref)	1.233 (0.461–3.300)	1.369 (0.544–3.442)	2.585 (1.062–6.292)	0.0150	0.7742
	≥50 years, adjusted OR (95% Cl)	1 (ref)	1.199 (0.953–1.507)	1.495 (1.200–1.862)	2.009 (1.627–2.480)	<0.0001	
Sex	Male, adjusted OR (95% Cl)	1 (ref)	1.165 (0.889–1.528)	1.347 (1.034–1.754)	1.929 (1.497–2.485)	<0.0001	0.5150
	Female, adjusted OR (95% Cl)	1 (ref)	1.390 (0.926–2.086)	1.986 (1.364–2.890)	2.522 (1.756–3.624)	<0.0001	
Smoking	Smoking, adjusted OR (95% Cl)	1 (ref)	1.214 (0.811–1.816)	1.698 (1.152–2.502)	1.771 (1.198–2.617)	0.0010	0.2195
	Does not smoke, adjusted OR (95% Cl)	1 (ref)	1.242 (0.946–1.630)	1.455 (1.123–1.886)	2.271 (1.778–2.901)	<0.0001	
Drinking	Drinking, adjusted OR (95% Cl)	1 (ref)	1.050 (0.708–1.557)	1.593 (1.097–2.312)	1.935 (1.338–2.798)	<0.0001	0.4963
	Does not drink, adjusted OR (95% Cl)	1 (ref)	1.351 (1.026–1.778)	1.511 (1.160–1.968)	2.225 (1.731–2.859)	<0.0001	

Adjustment factors include sex, age, smoking status, alcohol consumption, history of cerebral infarction, hypertension, atrial fibrillation, coronary heart disease, diabetes mellitus, WBC, FPG, LDL-C, Hcy, hs-CRP, hours of event onset, NIHSS score at onset, and mRS score before onset ≥3. Abbreviations: CI: confidence interval; Q: quartile; OR: odds ratio; mRS: modified Rankin Scale; NIHSS: National Institutes of Health Stroke Scale; SII: systemic immune-inflammation index; WBC: white blood cell; FPG: fasting plasma glucose; LDL-C: low-density lipoprotein cholesterol; Hcy: homocysteine; hs-CRP: high-sensitivity C-reactive protein.

### SII and secondary outcomes

**Figure 2:**
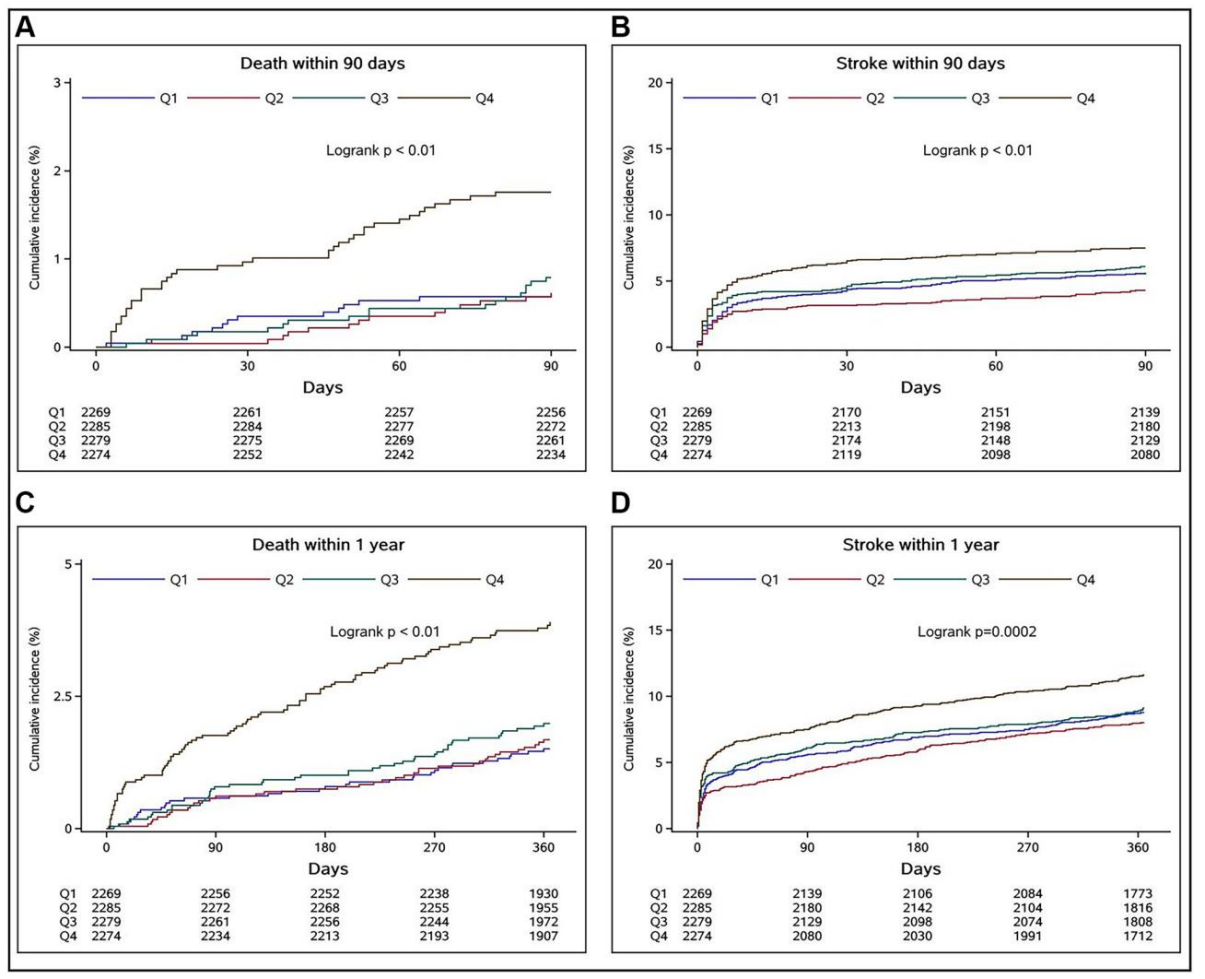
**Relationships between SII quartiles and cumulative incidence rates of all-cause deaths and recurrent strokes in stroke patients at 90-day and 1-year follow-up assessments.** (**A** and **B**) indicate the relationships of the SII quartiles with the cumulative incidence rates of all-cause death and recurrent stroke at the 90-day follow-up, respectively, whereas (**C** and **D**) show the respective outcomes at the 1-year follow-up.

During the 90-day and 1-year follow-up, the cumulative incidence rates of the secondary outcomes in different SII quartiles were represented with Kaplan–Meier curves (Figure 2). When the SII was high, the cumulative incidence of all-cause death and recurrent stroke during follow-up (log-rank test *P* < 0.01 for all outcomes) was also high.

Table 3 shows the data of secondary outcomes during the 90-day and 1-year follow-up, as well as the results of the Cox regression analysis between SII quartiles and secondary outcomes. Compared to Q1 of the SII, Q4 was associated with an increased risk of all-cause death (adjusted hazard ratio [HR], 2.409; 95% CI, 1.273–4.559) and recurrent stroke (adjusted HR, 1.279; 95% CI, 1.012–1.617) at the 90-day follow-up (all *P* < 0.05). Similarly, Q4 of the SII was associated with an increased risk of all-cause death (adjusted HR, 2.209; 95% CI, 1.474–3.311) and recurrent stroke (adjusted HR, 1.272; 95% CI, 1.054–1.536) at the 1-year follow-up (all *P* < 0.05).

**Table 3:** Multivariate Cox regression analysis of the relationships between SII and clinical outcomes of patients with acute ischemic stroke.

**Outcomes**	**Groups**	**Events, *n* (%)**	**Crude HR (95% CI)**	***P* value**	**Adjusted HR (95% CI)**	***P* value**
**90-day follow-up**						
Recurrent stroke 534 (5.9%)	Q1	127 (5.6)	1 (ref)		1 (ref)	
	Q2	98 (4.3)	0.762 (0.585–0.991)	0.0430	0.768 (0.590–1.001)	0.0505
	Q3	139 (6.1)	1.094 (0.860–1.392)	0.4624	1.081 (0.849–1.376)	0.5291
	Q4	170 (7.5)	1.355 (1.077–1.705)	0.0096	1.279 (1.012–1.617)	0.0397
All-cause death 85 (0.9%)	Q1	13 (0.6)	1 (ref)		1 (ref)	
	Q2	14 (0.6)	1.068 (0.502–2.272)	0.8644	1.175 (0.550–2.512)	0.6772
	Q3	18 (0.8)	1.378 (0.675–2.812)	0.3788	1.366 (0.667–2.797)	0.3938
	Q4	40 (1.8)	3.092 (1.654–5.781)	0.0004	2.409 (1.273–4.559)	0.0069
**1-year follow-up**						
Recurrent stroke 847 (9.3%)	Q1	198 (8.7)	1 (ref)		1 (ref)	
	Q2	182 (8.0)	0.907 (0.741–1.109)	0.3401	0.909 (0.743–1.112)	0.3549
	Q3	206 (9.0)	1.042 (0.857–1.266)	0.6819	1.023 (0.841–1.244)	0.8210
	Q4	261 (11.5)	1.348 (1.121–1.621)	0.0015	1.272 (1.054–1.536)	0.0122
All-cause death 205 (2.3%)	Q1	34 (1.5)	1 (ref)		1 (ref)	
	Q2	38 (1.7)	1.109 (0.698–1.762)	0.6608	1.185 (0.745–1.885)	0.4747
	Q3	45 (2.0)	1.318 (0.844–2.058)	0.2242	1.286 (0.822–2.011)	0.2707
	Q4	88 (3.9)	2.621 (1.764–3.893)	<0.0001	2.209 (1.474–3.311)	0.0001

Adjustment factors include sex, age, smoking status, alcohol consumption, history of cerebral infarction, hypertension, atrial fibrillation, coronary heart disease, diabetes mellitus, WBC, FPG, LDL-C, Hcy, hs-CRP, hours of event onset, NIHSS score at onset, and mRS score before onset ≥3. Abbreviations: CI: confidence interval; Q: quartile; HR: hazard ratio; mRS: modified Rankin Scale; NIHSS: National Institutes of Health Stroke Scale; SII: systemic immune-inflammation index; WBC: white blood cell; FPG: fasting plasma glucose; LDL-C: low-density lipoprotein cholesterol; Hcy: homocysteine; hs-CRP: high-sensitivity C-reactive protein.

## DISCUSSION

As the SII quartile increased, patients with acute ischemic stroke were more likely to have poor functional outcomes during the follow-up. The proportion of poor functional outcomes at 1-year follow-up was slightly lower than that at 90-day follow-up, suggesting that some patients were still recovering during this period, but the recovery was slow, and the proportion was low. To determine whether there were specific subpopulations whose characteristics were affected by inflammation, a stratified analysis was performed. Although age, smoking status, and alcohol consumption are risk factors [9–11] and female estrogen is a protective factor for stroke [12], we did not identify subgroups specifically being affected by these factors, indicating that the SII is relatively independent and stable (Table 2, *P* ≥ 0.10 for all interactions). Figure 2 shows that patients in the Q4 group had the highest cumulative incidence of all-cause death and recurrent stroke during the follow-up period (log-rank test *P* < 0.01 for all outcomes). Finally, the Cox regression analysis showed that Q4 of the SII at admission was an independent risk factor for all-cause death and recurrent stroke in patients with acute ischemic stroke during the 90-day and 1-year follow-up periods. Even if we adjusted our model for factors identified by previous studies as immune-inflammation markers that might affect prognosis, such as the high-sensitivity C-reactive protein level, our results still suggested that SII is closely related to short- and long-term prognosis of patients with acute ischemic stroke.

Previous studies have shown that the immune and inflammatory response after stroke is an important factor that affects patient prognosis [13, 14]. Neutrophils and lymphocytes are involved in the inflammatory and immune response, whereas platelets have a primary role in the thrombo-inflammation of stroke. As a new type of immune-inflammation index, the SII integrates neutrophils, platelets, and lymphocytes and can reflect the balance of the systemic immune response and inflammatory response [5]. Studies have also shown that neutrophils, which are the primary cells of the inflammatory response, are recruited in large numbers to participate in the immune-inflammatory response of post-stroke lesions [15, 16]. The massive production of platelets after a stroke is also involved in the pathological processes of infarct lesions [17]. The interaction between neutrophils and platelets exacerbates the destruction created by infarcts [18], causing a series of pathological reactions, such as the production of oxygen free radicals, exudation, and extracellular traps, which can induce thrombosis [19] and result in further damage to the ischemic brain tissue [7]. In a recent review by Langer et al., it was concluded that platelets mediate the immune-inflammatory response along with a variety of cells and can cause injury both inside and outside blood vessels, as well as to neurons [20]. These effects show that platelets are vital contributors to thrombotic inflammation and play a very important role in the immune-inflammatory response after stroke. Previous studies have shown that immunosuppression and decreased levels of lymphocytes after a stroke lead to a worse prognosis [21–23]. This explains how, in accordance with cellular-level pathophysiology, the SII can quite accurately reflect the systemic immune-inflammatory state and predict the prognosis of patients with stroke.

The SII can reveal the state of systemic immune-inflammatory response in patients with stroke and predict the development and prognosis of stroke. The primary advantage of this indicator is that it can be easily calculated from the results of routine blood tests that are mandatory upon hospital admission for every patient. To a certain extent, this research can provide evidence and guidance for clinicians in the treatment of stroke and prediction of its prognosis. The earlier high-risk patients are identified, the better the attention and care that will be given. This will improve the prognosis of patients with stroke and can minimize the burden of the disease on patients, their families, and the entire society. The main advantages of this study include its large sample size, multicenter nature, prospective demographics, and clinical follow-up data.

This study has some limitations. First, the study excluded patients with missing SII values and missing follow-up outcomes, which may have created a selection bias to some extent. Second, dynamic changes in the immune-inflammatory state were not considered in our data collection or analysis despite studies that have shown that the immune-inflammatory response state changes over time [24–26]. However, we strictly controlled the schedule of admission time and blood collection of patients and kept it as consistent as possible. Lastly, the study did not compare other indices, such as the neutrophil/lymphocyte ratio and platelet/lymphocyte ratio. However, the study can still provide good support and reference for clinical and basic research.

In conclusion, our research shows that patients with higher SIIs after an acute ischemic stroke were more likely to have poor functional outcomes at the 90-day and 1-year follow-ups. Furthermore, patients with higher SIIs were more likely to experience all-cause death and recurrent stroke.

## MATERIALS AND METHODS

### Study population

Data were derived from the CNSR-III. The CNSR-III is a nationwide, multicenter, prospective registry of patients with ischemic stroke and transient ischemic attack in China. The database includes complete imaging and biomarker data [27]. The study included patients in the CNSR-III with ischemic stroke who were admitted to a hospital within 72 h of onset but excluded the following: 1) patients with cancer and peripheral thrombosis; 2) patients with active infections or those who took antibiotics or immunosuppressive agents 2 weeks before admission and during hospitalization; and 3) patients with missing SII and follow-up data (shown in Supplementary Figure 1).

### Data collection

The patients’ baseline data included their age, sex, smoking status, alcohol consumption, medical history, time from stroke onset to admission, NIHSS score at admission, and laboratory data. All the above data were collected by trained research coordinators following a standard data collection protocol. The coordinators identified the eligible patients, obtained informed consent, enrolled consecutive patients, and collected data through face-to-face interviews with the patients. Fasting venous blood was withdrawn from all patients within 24 h after admission, and an automatic routine blood analysis was performed. We calculated the SII for each patient based on the results of the routine blood analysis using the formula: neutrophils × platelets/lymphocytes.

### Outcome assessment

According to the CNSR-III research protocol, researchers conducted face-to-face 90-day and 1-year follow-ups of patients. At the follow-up, the mRS score was used to assess the functional outcomes of the patients. The primary outcome was poor functional outcome, defined as 3≤mRS score≤6, where 6 points indicated death. The secondary outcome was the incidence of all-cause death and recurrent stroke.

### Statistical analysis

Categorical variables were described as percentages and continuous variables as either means and standard deviations or medians and interquartile ranges. According to the quartile of the SII value, the eligible study participants were divided into four groups and compared using the χ^2^ test for categorical variables. The Kruskal–Wallis test was used to compare continuous variables. Logistic regression was used to analyze the relationships between the SII quartile groups and poor functional outcome at the follow-up points, with SII Q1 as the reference group. In addition, we conducted stratified analyses by trend and interaction tests. The cumulative incidence of adverse events was described using the Kaplan–Meier curves and compared using the log-rank test. The Cox regression analysis was used to explore the HR between the SII quartiles and adverse events at different follow-up points. The selection of adjustment variables in the adjustment model of logistic regression analysis and Cox regression analysis was derived from the variables with significant differences in univariate analysis or variables considered to be clinically significant. A two-tailed *P* value < 0.05 was considered statistically significant. All statistical analyses were completed using the SAS software (version 9.4; SAS Institute, Inc., Cary, NC, USA).

### Data availability statement

Part of the data in this article is provided in the supplementary material. Other anonymized data not published within this article will be made available to any qualified investigator by request from the corresponding author.

## Supplementary Materials

Supplementary Figure 1

Supplementary Table 1
