# Supplementary material for: Correlation of the systemic immune-inflammation index with short- and long-term prognosis after acute ischemic stroke
Source: Aging (Albany NY). 2022 Aug 19;14(16):6567–78. doi: 10.18632/aging.204228 (PMC9467411; doi:10.18632/aging.204228)
Supplement: Supplementary Figure 1 [file aging-14-204228-s001.pdf]

SUPPLEMENTARY FIGURE

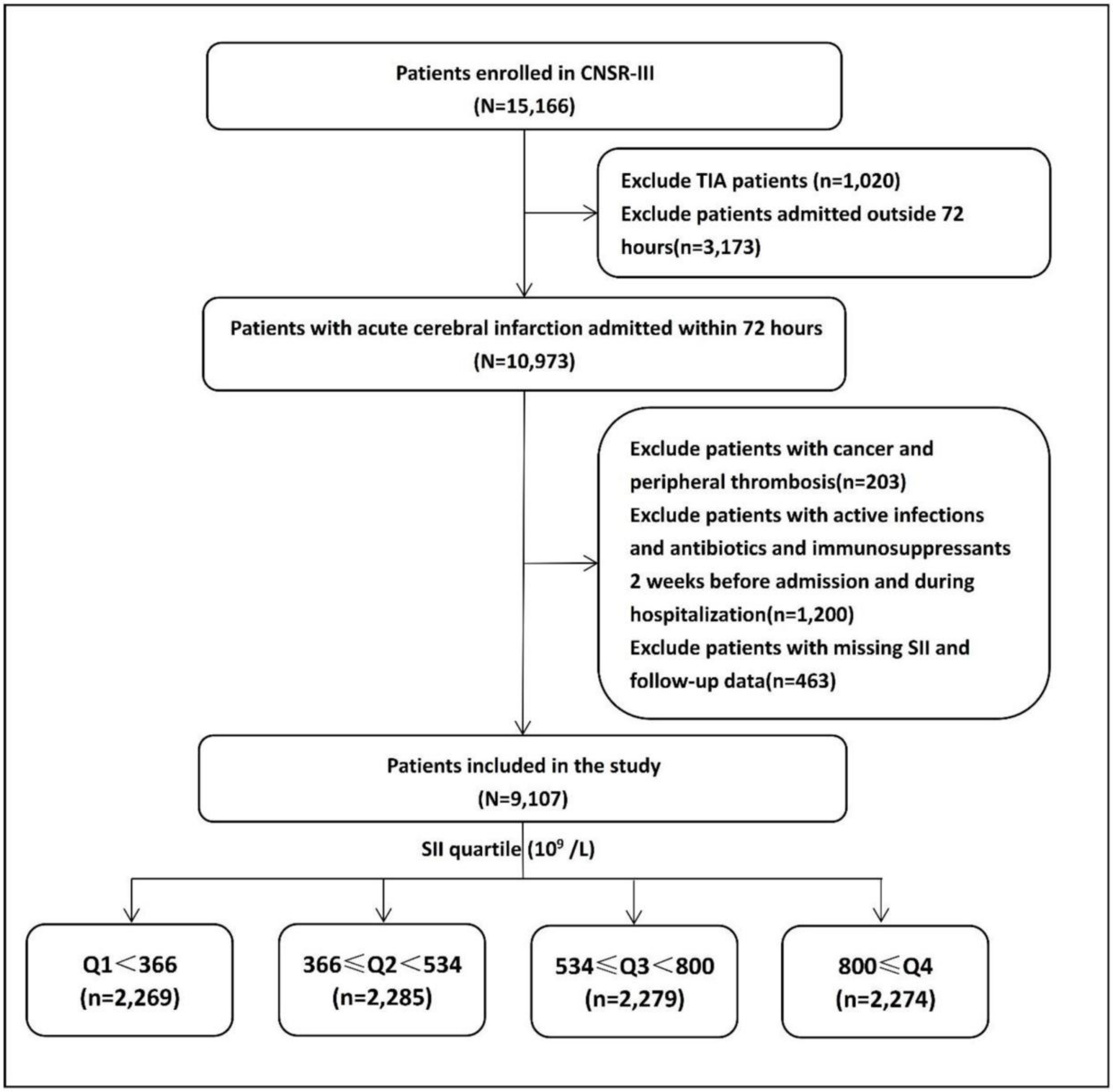

Supplementary Figure 1. Flow chart of inclusion and exclusion criteria for the study population. Based on the inclusion and exclusion criteria (see Methods for details), we selected the population to be studied. Abbreviations: CNSR-III: China National Stroke Registry III; SII: systemic immune inflammation index (neutrophil × platelet/lymphocyte, 10^9/L).
