# Supplementary material for: Correlation of the systemic immune-inflammation index with short- and long-term prognosis after acute ischemic stroke
Source: Aging (Albany NY). 2022 Aug 19;14(16):6567–78. doi: 10.18632/aging.204228 (PMC9467411; doi:10.18632/aging.204228)
Supplement: Supplementary Table 1 [file aging-14-204228-s002.pdf]

SUPPLEMENTARY TABLE

Supplementary Table 1. Baseline comparison of patients with included and excluded.

Characteristics	Included patients	Excluded patients	*P*-value
**Demography and clinical features**			
Age(yr), mean ± SD	61.88 ± 11.11	62.76 ± 11.57	<.0001
Male, *n* (%)	6343 (69.7)	4021 (66.4)	<.0001
Smoking, *n* (%)	3317 (36.4)	2095 (34.6)	0.0201
Drinking, *n* (%)	3553 (39.0)	2162 (35.7)	<.0001
**Medical history**			
Ischemic stroke, *n* (%)	1850 (20.3)	1299 (21.4)	0.0943
Coronary heart diseases, *n* (%)	914 (10.0)	694 (11.5)	0.0055
Atrial fibrillation, *n* (%)	592 (6.5)	427 (7.1)	0.1876
Hypertension, *n* (%)	5717 (62.8)	3777 (62.3)	0.5843
Diabetes mellitus, *n* (%)	2126 (23.3)	1384 (22.8)	0.4722
Hypercholesterolemia, *n* (%)	696 (7.6)	495 (8.2)	0.2372
**Hours of event onset, median (IQR)**	10 (3–25)	20 (3–76)	<.0001
**NIHSS at admission, median (IQR)**	3 (2–6)	3 (1–6)	<.0001
**mRS score before onset ≥3, *n* (%)**	370 (4.1)	287 (4.7)	0.0458
**Laboratory tests**			
WBC, (10^9/L)	7.24 ± 2.21	7.91 ± 40.32	0.0561
Neutrophil, (10^9/L)	4.85 ± 2.23	4.99 ± 2.32	0.0463
Platelet, (10^9/L)	217.60 ± 63.37	217.32 ± 65.61	0.2199
Lymphocyte, (10^9/L)	1.81 ± 0.79	1.78 ± 0.84	0.0106
FPG (mmol/L)	6.46 ± 2.62	6.34 ± 2.49	0.0210
LDL (mmol/L)	2.56 ± 1.04	2.55 ± 1.06	0.1947
HCY (µ mol/L)	18.93 ± 12.26	19.24 ± 12.47	0.0425
hs-CRP (mg/L）	5.67 ± 18.22	10.74 ± 33.76	<.0001

Abbreviations: SD: standard deviation; IQR: interquartile range; NIHSS: National Institutes of Health Stroke Scale; Q: quartile; mRS: modified Rankin Scale; WBC: white blood cell; FPG: fasting plasma glucose; LDL: low-density lipoprotein; Hcy: homocysteine; hs-CRP: high sensitivity C reactive protein.
